# Preparation of Am-MSN/PVDF Mixed Matrix Membranes for Enhanced Removal of Reactive Black 5

**DOI:** 10.3390/membranes15020042

**Published:** 2025-02-01

**Authors:** Jihao Zuo, Mengkang Lu, Jinting Cai, Ruopeng Lan, Xinjuan Zeng, Cailong Zhou

**Affiliations:** 1 Key Laboratory of Green Prevention and Control on Fruits and Vegetables in South China of Ministry of Agriculture and Rural Affairs, School of Chemistry and Chemical Engineering, Zhongkai University of Agriculture and Engineering, Guangzhou 510225, China; zuojihao@zhku.edu.cn (J.Z.); cjinting1002@163.com (J.C.); m18475704550@163.com (R.L.); 2 Key Laboratory of Green Surface Technology and Functional Coatings for Materials, China National Light Industry, School of Materials and Energy, Foshan University, Foshan 528000, China; lmk484076924@163.com; 3 School of Chemistry and Chemical Engineering, Chongqing University, Chongqing 400044, China

**Keywords:** polyvinylidene difluoride (PVDF), mesoporous silica, mixed matrix membrane, adsorption, dye removal

## Abstract

The discharge of large volumes of textile dyeing wastewater, characterized by poor biodegradability and high toxicity, poses severe threats to the environment. In this study, polyvinylidene difluoride (PVDF) membranes were prepared using the nonsolvent-induced phase separation (NIPS) method, with porous amino-functionalized mesoporous silica nanoparticles (Am-MSNs) mixed into the casting solution to fabricate the Am-MSN/PVDF mixed matrix membranes. By varying the amount of Am-MSNs added, the microstructure and overall performance of the membranes were comprehensively analyzed. The results demonstrated that the addition of Am-MSNs significantly enhanced the hydrophilicity of the membranes. The high specific surface area and amino groups of Am-MSNs facilitated interactions with dye molecules, such as Reactive Black 5 (RB5), through hydrogen bonding, electrostatic attraction, and physical adsorption, resulting in a marked improvement in RB5 rejection rates. Static adsorption tests further validated the superior adsorption capacity of the Am-MSN/PVDF mixed matrix membranes for RB5. Additionally, the nanoscale mesoporous structure of Am-MSNs enhanced the mechanical strength of the membranes. The synergistic effects of the mesoporous structure and amino groups significantly increased the efficiency and stability of the Am-MSN/PVDF mixed matrix membranes in dye removal applications, providing an effective and sustainable solution for the treatment of dye-contaminated wastewater.

## 1. Introduction

With the rapid development of the dyeing industry, a significant amount of dye wastewater is directly discharged into water bodies, causing severe harm to the aquatic environment [1,2,3]. Once introduced into natural water systems, dyes not only inhibit the growth and development of aquatic organisms but also contribute to the eutrophication of water bodies. Additionally, the infiltration of dyes into groundwater poses serious health risks to humans [4,5,6]. Therefore, the efficient removal of dyes from wastewater has emerged as a pressing environmental and public health concern. Due to their high toxicity and resistance to degradation, dyes present persistent challenges for wastewater treatment processes.

The treatment of textile dyeing wastewater can be divided into three main methods, which are chemical, biological, and physical approaches. Chemical treatments encompass techniques such as electrochemical methods, advanced oxidation processes, and coagulation–flocculation [7,8,9]. Biological treatments include aerobic and anaerobic methods [10,11,12], while physical treatments primarily involve adsorption, ultrasound, and membrane processes [13,14,15]. Among these, membrane filtration stands out as an environmentally favorable option compared to traditional chemical treatments, as it avoids the production of harmful secondary pollutants, such as sludge or chemical by-products [16,17]. In contrast, biological treatment methods face significant limitations, including prolonged microbial cultivation periods, high energy demands, elevated operational costs, and considerable sludge generation. Membrane processes provide high separation efficiency and are capable of effectively removing dissolved substances, suspended solids, dyes, bacteria, and other pollutants from wastewater [18,19]. Furthermore, the pore size of membrane materials can be tailored to selectively remove pollutants of varying molecular sizes, enabling efficient and targeted separation [20,21].

In recent years, polymeric separation membranes have gained significant attention for dye removal and have seen rapid advancements [22,23]. Among various polymer materials, PVDF has emerged as a preferred choice for membrane fabrication due to its exceptional mechanical strength, thermal stability, chemical resistance, and film-forming properties [24,25]. It is extensively used in the production of ultrafiltration and microfiltration membranes. However, due to the inherently low surface energy of PVDF, the material exhibits strong hydrophobicity, which results in low permeation flux during filtration processes [26,27]. Moreover, its weak interactions with dye molecules hinder the efficient removal of dyes. Enhancing the hydrophilicity of PVDF membranes is crucial for expanding the application of polymeric membranes in textile dyeing wastewater treatment [28,29]. The blending method is widely employed to improve the hydrophilicity of PVDF membranes, offering the advantage of integrating material preparation and modification into a single step, without the need for pre-treatment or post-treatment. Through this method, PVDF and modifying agents coexist on the membrane surface and within its pores, significantly improving the membrane’s physicochemical properties. Inorganic nanoparticles, such as graphene oxide (GO), titanium dioxide (TiO_2_), and silicon dioxide (SiO_2_), are commonly utilized as hydrophilic modifiers for PVDF membranes [29,30,31]. Despite the simplicity of the blending modification process, it requires stringent operational conditions and the careful selection of suitable additives. If the additives aggregate or leach out, uneven membrane formation may occur, adversely affecting the membrane’s morphology and performance [32].

**Scheme 1:**
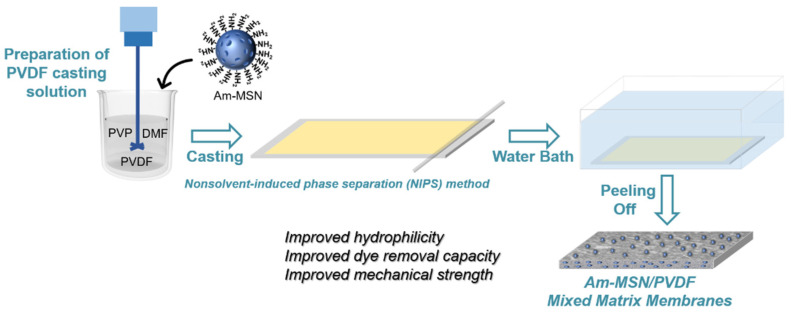
Schematic illustration of the fabrication process for the Am-MSN/PVDF mixed matrix membranes.

In this study, amino-functionalized mesoporous silica (Am-MSN) was utilized as a nanofiller and mixed into a PVDF casting solution to fabricate the Am-MSN/PVDF mixed matrix membranes via the nonsolvent-induced phase separation (NIPS) method (Scheme 1). Reactive Black 5 (RB5), a water-soluble acidic dye commonly used in textile dyeing, was selected as the model pollutant for removal experiments. RB5 possesses a complex molecular structure comprising multiple benzene rings, azo groups, and sulfonic acid groups, which contribute to its strong interactions with fibers, high environmental persistence, and resistance to degradation, particularly in aquatic systems. The inclusion of Am-MSNs within the PVDF matrix offers two major benefits. First, it enhances the membrane’s hydrophilicity and mechanical strength. Second, the high specific surface area of Am-MSNs provides abundant adsorption sites for dye molecules through mechanisms such as hydrogen bonding, electrostatic attraction, and physical adsorption through its mesoporous structure. These combined effects significantly enhance the efficiency and stability of the Am-MSN/PVDF mixed matrix membranes in dye removal applications, offering promising potential for industrial wastewater treatment and water resource recovery.

## 2. Experimental

### 2.1. Materials

Original mesoporous silica nanoparticles (MSNs) were obtained from Zhongke Keyou Nano Technology Co., Ltd. (Beijing, China). Polyvinylidene difluoride (PVDF, average Mw~400,000, powder), polyvinylpyrrolidone (PVP, K88-96, Mw = 1,300,000), and N,N-dimethylformamide (DMF, 99.5%) were procured from Macklin Biochemical Technology Co., Ltd. (Shanghai, China). Toluene, 3-aminopropyltriethoxysilane (KH-550), anhydrous ethanol, and Reactive Black 5 (RB5) were supplied by Aladdin Biochemical Technology Co., Ltd. (Shanghai, China). Deionized water used in all experiments was sourced from the laboratory’s water purification system.

### 2.2. Synthesis of Am-MSNs

To prepare amino-functionalized mesoporous silica nanoparticles (Am-MSNs), 1 g of original MSNs was dispersed in 50 mL of toluene in a three-neck flask and subjected to ultrasonication for 5 min. Separately, 1 mL of 3-aminopropyltriethoxysilane (KH-550) was mixed with 5 mL of toluene, and the resulting solution was gradually added to the MSN–toluene mixture. The combined solution was stirred uniformly at 50 rpm for 12 h to ensure a complete reaction. Following the reaction, the modified MSNs was washed three times with deionized water and ethanol in sequence and then vacuum-dried at 50~70 °C for 24 h. The dried product was thoroughly ground into a fine powder, yielding the amino-functionalized mesoporous silica nanoparticles, referred to as Am-MSNs.

### 2.3. Preparation of Am-MSN/PVDF Mixed Matrix Membranes

In a single-neck round-bottom flask, 4 g of polyvinylpyrrolidone (PVP, 8 wt%), 11 g of polyvinylidene difluoride (PVDF, 22 wt%), and 35 g of N, N-dimethylformamide (DMF, 70 wt%) were combined and stirred thoroughly until a homogeneous mixture was achieved. Subsequently, specific amounts of Am-MSNs (0.3 g and 0.6 g) were added to the mixture, which was mechanically stirred at 80 °C for 12 h to produce a uniform casting solution. After stirring, the casting solution was degassed in an oven at 60 °C for 8 h to remove any trapped air bubbles. A specified amount of the degassed solution was then poured in a strip onto a smooth glass plate and spread uniformly using a scraper with a thickness setting of 250 µm. Immediately following the casting process, the glass plate was immersed in a coagulation bath filled with deionized water to induce phase separation. Once the membrane solidified, it was carefully peeled off the glass plate and transferred into fresh deionized water. The water was replaced every 8 h to ensure complete solvent exchange.

For comparison, PVDF membranes without Am-MSNs and those incorporating original MSNs were prepared using the same casting procedure. Table 1 listed the detailed preparation conditions for all prepared membranes.

### 2.4. Characterization

The structure and morphology of nanoparticles and membranes were analyzed using scanning electron microscopy (SEM, Hitachi S4800, Tokyo, Japan). The microstructure of nanoparticles was further examined through transmission electron microscopy (TEM, JEOL JEM-2100F, Tokyo, Japan). The surface functional groups of nanoparticles and membranes were characterized using a Fourier transform infrared spectrometer (FTIR, PERKINELMER Spectrum 100, Waltham, USA) and an attenuated total reflection Fourier transform infrared spectrometer (ATR-FTIR, PERKINELMER Spectrum 100, Waltham, USA), respectively. The thermal stability of membranes was assessed using thermogravimetric analysis (TG, Mettler-Toledo, Zurich, Switzerland). The polymorphic structure of membranes was determined via wide-angle X-ray diffraction (WAXD, D/max Rapid II, Tokyo, Japan) with Cu Kα radiation, scanned over a range of 5° to 80°. The contact angles of water droplets on the membranes surface were analyzed using a contact angle meter (Theta Lite, Gothenburg, Sweden). The stress–strain curves of membranes were evaluated using a microcomputer-controlled electronic universal testing machine (Lishi Instruments, Shanghai, China).

### 2.5. Crystallinity Test

The thermal property and crystallinity of the membranes were analyzed using a DSC system (DSC Q100, TA Instruments, USA) under a nitrogen atmosphere. The heating rate was set to 5 °C/min, with a temperature range from 25 °C to 280 °C. In the DSC analysis, the melting temperature (*T_m_*) was determined from the peak of the endothermic curve, while the melting enthalpy (Δ*H_f_*) was calculated by integrating the area under the peak. Crystallinity (*X_c_*) was then calculated using Equation (1).

(1) $$X_{c}\left(\%\right)=\frac{{\Delta H}_{f}}{{\Delta H}_{f}^{*}}\times 100\%$$

where Δ*H_f_* represents the melting enthalpy of pure PVDF (104.7 J/g) and ΔH_f_^*^ refers to the melting enthalpy of the membrane being tested.

### 2.6. Porosity Test

The porosity (*ε*, %) of the PVDF membrane samples was determined using the gravimetric method. A dry membrane with specified dimensions (1 × 2 cm^2^) was weighed to record its dry mass (*M*_d_). The membrane was then immersed in 2-propanol for 24 h to ensure complete saturation. After immersion, the membrane was removed, and excess solvent on its surface was carefully blotted using lens paper. The membrane was subsequently weighed to determine its wet mass (*M*_w_). The porosity of the membrane was calculated using the following Equation (2):

(2) $$\varepsilon =\frac{(Mw-Md)\times {\;\rho }_{poly}}{\rho_{poly}\times Mw+(\rho_{IPA}-\rho_{poly})\times Md}\times 100\%$$

where *ε* represents the membrane porosity, *M*_w_ is the wet membrane mass (kg), *M*_d_ is the dry membrane mass (kg), *ρ_IPA_* is the density of 2-propanol (kg m^−3^), and *ρ_PVDF_* is the density of the PVDF polymer (kg m^−3^).

### 2.7. Dye Filtration Test

The permeation and filtration experiments in this study were conducted using a custom-made dead-end filtration setup. The effective filtration area of the membrane was 0.785 cm^2^. Permeation tests were carried out at 25 °C under a pressure of 0.4 MPa. During the pure water flux measurement, the membrane was pre-pressurized at 0.4 MPa using pure water until the permeation flux stabilized. The pure water flux (*J*_w_) was then calculated using Equation (3):

(3) $$J_{w}\;=\frac{V}{S\times t}$$

where *J*_w_ is the pure water flux (L m^−2^ h^−1^), *V* is the permeated volume (L), *S* is the membrane area (m^2^), and *t* is the filtration time (h).

To evaluate the rejection rate of the RB5 dye solution, the membrane was first pre-pressurized at 0.4 MPa using a 10 ppm RB5 dye solution until the permeation flux stabilized. The volume of the dye solution fed in a single operation is 100 mL. The permeation flux for the feed solution was also calculated using the same equation. Once a stable permeation flux was achieved, the feed solution was replaced with a 30 ppm RB5 dye solution. After filtering 5 mL of the dye solution, the permeate was collected, and the rejection rate *R* of the membrane for the RB5 dye was calculated using Equation (4):

(4) $$R=\frac{C_{1}-C_{2}}{C_{1}}$$

where *R* is the RB5 rejection rate, *C*_1_ is the concentration of the feed solution (ppm), and *C*_2_ is the concentration of the permeate (ppm). *C*_1_ and *C*_2_ were determined by measuring the absorbance of the feed and permeate solutions at a wavelength of 597 nm using a UV–visible spectrophotometer (Shimadzu UV-3600Plus, Kyoto, Japan).

### 2.8. Static Adsorption Test

A static adsorption experiment was conducted using membranes of a specific size (1 × 2 cm^2^). The membrane samples were immersed in 10 mL of an RB5 azo dye solution with an initial concentration of 30 mg/L, with constant magnetic stirring for 24 h. The equilibrium adsorption capacity of the membrane was calculated using Equation (5):

(5) $$Q_{e\;}=\frac{{(C}_{0}-C_{e})\times V}{m}$$

where *Q_e_* is the equilibrium adsorption capacity of the dye (mg/g), *C*_0_ is the initial dye concentration (mg L^−1^), *C_e_* is the equilibrium dye concentration after contact with the membrane (mg L^−1^), *V* is the volume of the solution (L), and m is the mass of the membrane or the mass of the membrane containing Am-MSNs (g).

## 3. Results and Discussion

### 3.1. Surface Composition Analysis of Nanoparticles and Membranes

**Figure 1:**
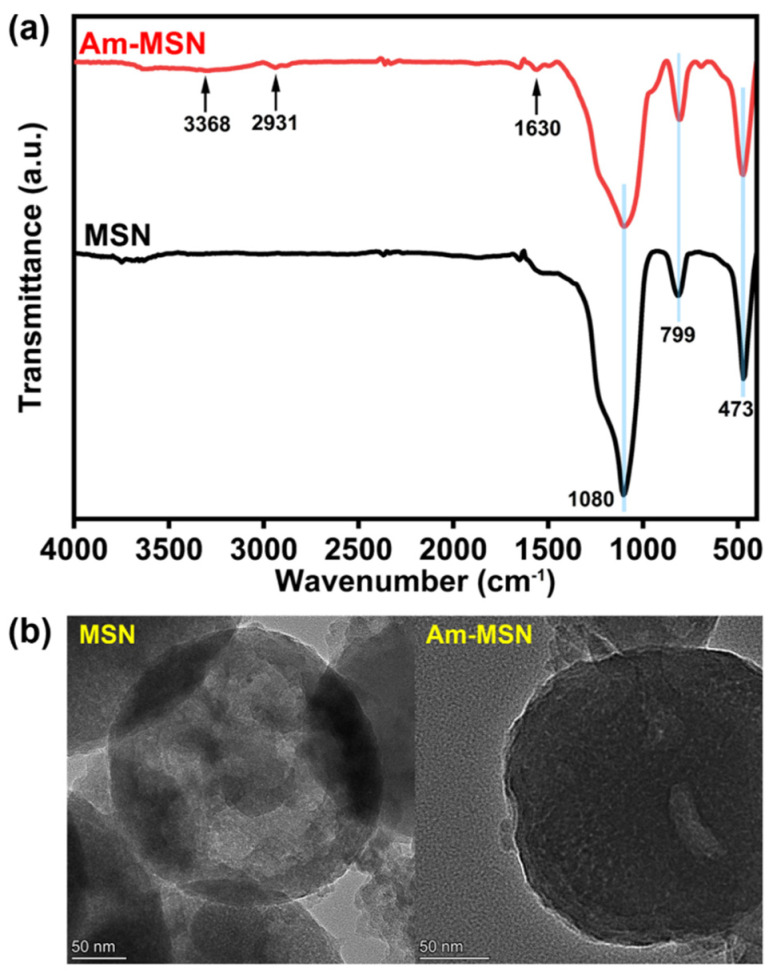
(**a**) The FTIR spectra and (**b**) TEM images of MSNs and Am-MSNs.

Figure 1a illustrates the Fourier transform infrared (FTIR) spectra of the original MSNs and Am-MSNs. The peaks observed at approximately 473, 799, and 1080 cm^−1^ are characteristic of the Si-O-Si bonds in silica [33,34]. In comparison with the original MSNs, the Am-MSNs display additional absorption peaks at 1630 cm^−1^ and 3368 cm^−1^, corresponding to the amide I band and the N-H stretching vibration, respectively [35,36]. Furthermore, the peak at 2931 cm^−1^, indicative of the typical C-H stretching vibration [37], originates from the amino source KH550, a silane coupling agent containing organic chains. These FTIR results confirm the successful amino-functionalization of mesoporous silica.

Figure 1b presents the TEM images of the MSNs and Am-MSNs. The MSNs exhibit a spherical morphology with a size range of 200~500 nm, and their contours are clearly defined. Following amino-functionalization, the surface of the Am-MSNs becomes noticeably rougher [38], with increased irregularity in the pore wall boundaries. This structural alteration can be attributed to the grafting of amino chains onto the silica surface.

**Figure 2:**
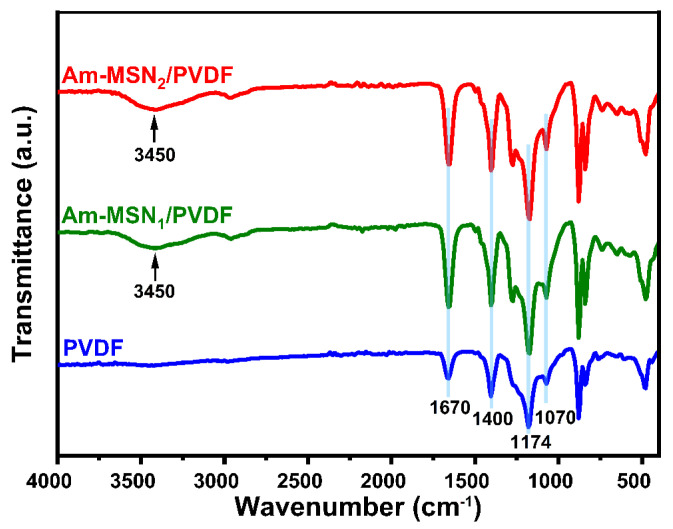
The ATR-FTIR spectra of pure PVDF, Am-MSN_1_/PVDF, and Am-MSN_2_/PVDF membranes.

Figure 2 displays the attenuated total reflection Fourier transform infrared (ATR-FTIR) spectra of pure PVDF, Am-MSN_1_/PVDF, and Am-MSN_2_/PVDF membranes. The peak observed at 1670 cm^−1^ is attributed to the C=O stretching vibration of the pyrrolidone group in PVP. The peaks at 1400 cm^−1^ and 1174 cm^−1^ correspond to the stretching and deformation vibrations of the C-H bond and the stretching vibration of the C-F bond, respectively [39]. Meanwhile, the peak at 1070 cm^−1^ is assigned to the stretching vibration of the C-C bond. Notably, after doping with Am-MSNs, the PVDF membranes exhibit characteristic amino peaks in the range of 3300~3500 cm^−1^ [40], which provides direct evidence of the successful incorporation of Am-MSNs into the membrane matrix.

**Figure 3:**
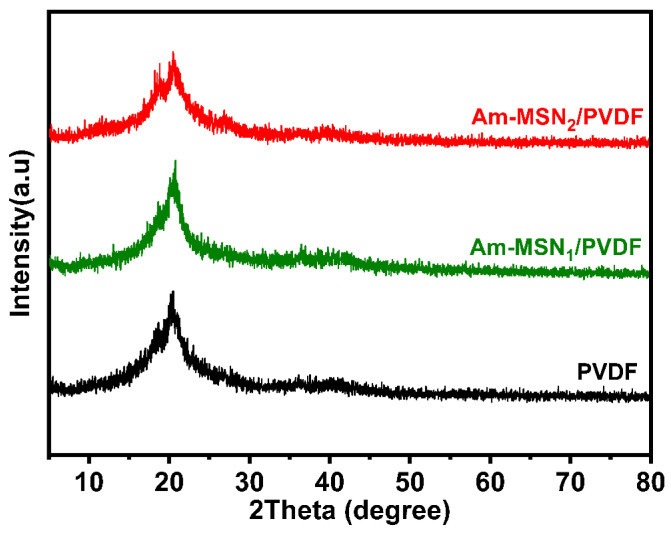
XRD patterns of the pure PVDF, Am-MSN_1_/PVDF, and Am-MSN_2_/PVDF membranes.

**Figure 4:**
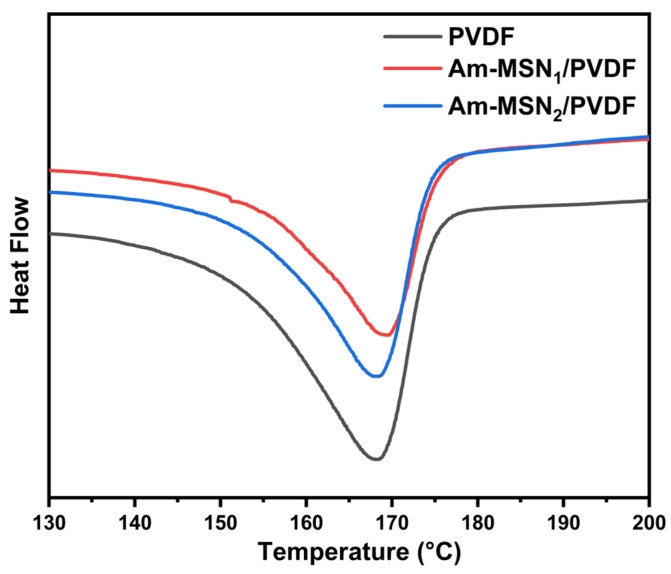
The DSC curves of the pure PVDF, Am-MSN_1_/PVDF, and Am-MSN_2_/PVDF membranes.

Figure 3 illustrates the XRD patterns of pure PVDF, Am-MSN_1_/PVDF, and Am-MSN_2_/PVDF membranes. The diffraction peaks at 18.38°, 36.82°, and 40.58° are associated with the α-phase of PVDF, while the peak at 20.44° corresponds to the β-phase [2,41]. A comparison of the XRD patterns for the Am-MSN/PVDF mixed matrix membranes reveals that the diffraction peaks at 18.38° (α-phase) and 20.44° (β-phase) are retained, though their integrated areas decrease significantly following the incorporation of Am-MSNs [42]. The thermal characteristics and crystallinity of the PVDF membranes were evaluated through DSC analysis, with the results presented in Figure 4. Table 2 also shows that, compared to the pure PVDF membrane, the melting temperatures of the Am-MSN_1_/PVDF and Am-MSN_2_/PVDF membranes are higher, indicating that the introduction of Am-MSNs altered the crystallization structure or thermal stability of the PVDF matrix. This can be attributed to the role of Am-MSNs as nucleating agents for PVDF, promoting more uniform and ordered crystal growth. Consequently, the thermodynamic stability of the crystalline regions in PVDF is enhanced, requiring a higher temperature for melting. Additionally, interactions between the Am-MSNs and PVDF polymer chains, potentially through hydrogen bonding or van der Waals forces, may restrict the mobility of the molecular chains, thereby increasing the energy barrier for melting [43]. These factors collectively contribute to the improved thermal stability and higher melting temperature of the Am-MSN_1_/PVDF and Am-MSN_2_/PVDF membranes. Moreover, the crystallinity of the composite membranes is higher than that of the pure PVDF membrane. This improvement can be attributed to the role of Am-MSNs as nucleation sites for PVDF crystallization. These nanoparticles facilitate better molecular alignment and denser packing of the polymer chains, thereby enhancing crystal stability [44]. In summary, the incorporation of Am-MSNs enhances the crystallinity of the PVDF matrix, while also improving the thermal stability and mechanical properties of the composite membranes. These findings suggest that the inclusion of Am-MSNs can effectively modulate the crystallization behavior of the membranes, potentially enhancing their functional performance.

**Figure 5:**
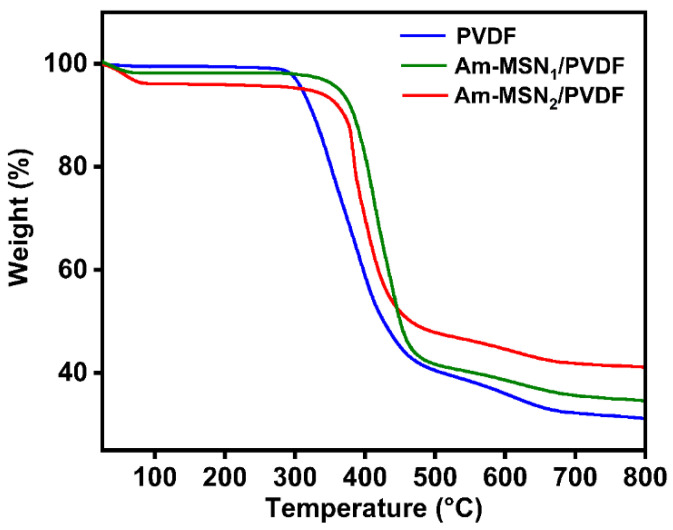
TG curves of the pure PVDF, Am-MSN_1_/PVDF, and Am-MSN_2_/PVDF membranes.

Figure 5 presents the TGA curves of the pure PVDF, Am-MSN_1_/PVDF, and Am-MSN_2_/PVDF membranes. In the initial stage of the thermogravimetric analysis, all samples exhibit a noticeable weight loss at lower temperatures (below approximately 200 °C). This weight loss is primarily attributed to the evaporation of physically adsorbed and chemically bound water within the membranes [45]. As the temperature exceeds 200 °C, a second stage of weight loss is observed, corresponding to the degradation of the polymer backbone and the cleavage of chemical bonds during the pyrolysis process. At temperatures above 500 °C, further weight loss occurs, primarily due to the decomposition of residual organic materials, such as pyrolysis by-products. For both pure PVDF and Am-MSN/PVDF mixed matrix membranes, the rate of weight loss gradually decreases and eventually stabilizes at higher temperatures. The thermal analysis results indicate that the incorporation of Am-MSNs significantly enhances the thermal stability of the PVDF membranes. Notably, the Am-MSN_2_/PVDF membrane exhibits a considerably higher decomposition temperature compared to the pure PVDF membrane. This enhancement is attributed to the strong physical and chemical interactions between the Am-MSNs and the PVDF polymer chains, which improve the thermal stability of the membrane matrix [46].

**Figure 6:**
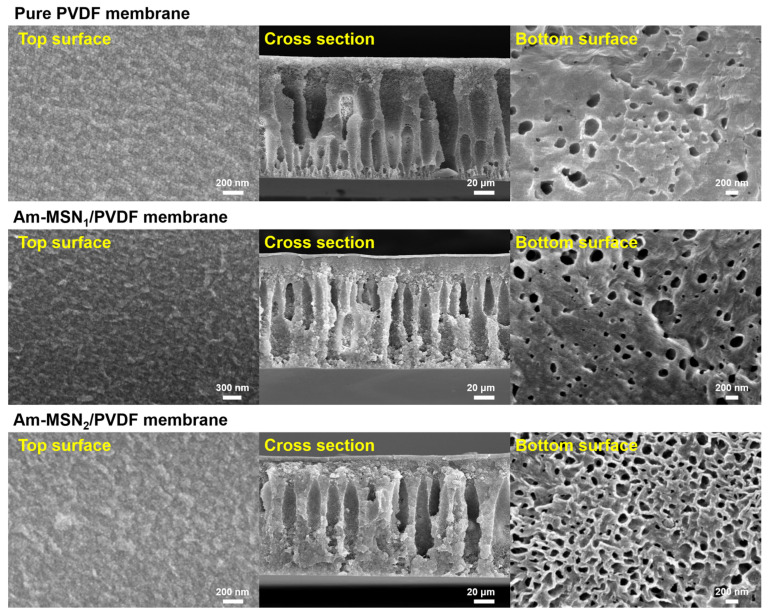
SEM images of the top surface, cross-section, and bottom surface morphologies of the pure PVDF, Am-MSN_1_/PVDF, and Am-MSN_2_/PVDF membranes.

The microstructures of the upper surface, cross-section, and lower surface of the pure PVDF, Am-MSN_1_/PVDF, and Am-MSN_2_/PVDF membranes were analyzed using the SEM images shown in Figure 6. The upper surface of the pure PVDF membrane exhibits a smooth and uniform morphology, lacking noticeable pores or particles, indicative of a dense and flat membrane structure. The cross-sectional view reveals vertically aligned finger-like pores with a large, orderly configuration. The dense top skin layer transitions into a more porous bottom layer. This arrangement enhances permeability while maintaining mechanical strength. The lower surface displays irregularly shaped macropores with a random distribution and sparse pore density [47]. The incorporation of Am-MSNs into the membrane matrix alters the surface and structural features. The upper surface of the Am-MSN_1_/PVDF membrane becomes noticeably rougher due to the irregular aggregation of Am-MSNs during the phase separation process. This surface roughness may improve the membrane’s wettability and interfacial interactions. The cross-sectional view shows that the finger-like pore structure is retained, with a slight increase in pore size and rougher pore walls, potentially enhancing permeability. The lower surface of the Am-MSN_1_/PVDF membrane exhibits a greater number of pores, with more varied pore sizes and thicker pore walls, suggesting that the addition of Am-MSNs promotes pore formation, increases specific surface area, and enhances adsorption properties. When the Am-MSN content is doubled, the Am-MSN_2_/PVDF membrane exhibits further changes. Its upper surface displays denser nanoscale particle structures and increased surface roughness compared to the Am-MSN_1_/PVDF membrane. In the cross-sectional view, the finger-like pores remain dominant, but their size decreases slightly, and the pore walls become denser and smoother. Near the bottom region, the number of pores increases, with a more uniform pore distribution. These structural modifications imply that a higher Am-MSN content improves the membrane’s structural integrity and may enhance its overall performance [40,48].

Table 3 summarizes the porosity of the pure PVDF, Am-MSN_1_/PVDF, and Am-MSN_2_/PVDF membranes. The results indicate that the Am-MSN/PVDF mixed matrix membranes exhibit a significant increase in porosity compared to the pure PVDF membrane. This enhancement is attributed to the highly ordered mesoporous structure and large specific surface area of the amino-functionalized silica particles. During the membrane formation process, the mesoporous silica particles, when incorporated into the PVDF casting solution, disperse uniformly and actively contribute to the development of the membrane’s porous structure. The inherent porosity of the silica particles introduces additional voids and pores within the PVDF matrix, thereby increasing the overall porosity. Furthermore, the size and distribution of the silica particles play a critical role in modifying the pore size and structure, which optimizes the membrane’s flux and selectivity. In the NIPS process, PVDF and the solvent undergo phase separation upon contact with the non-solvent, forming a porous structure. The incorporation of amino-functionalized mesoporous silica enhances this phase separation process through a “seeding effect” or interactions with PVDF’s molecular chains. These interactions accelerate the phase separation process, promoting the formation of additional voids and pores within the membrane structure and further increasing its porosity [49,50,51].

Table 4 summarizes the semi-quantitative elemental composition of the pure PVDF, Am-MSN_1_/PVDF, and Am-MSN_2_/PVDF membranes based on EDS analysis. The pure PVDF membrane primarily comprises fluorine (F) and carbon (C), consistent with its composition as polyvinylidene difluoride. Small amounts of nitrogen (N) detected are likely attributed to residual DMF and PVP used during membrane preparation, while oxygen (O) may result from moisture absorption or trace water remaining in the membrane during storage or processing. In the Am-MSN_1_/PVDF membrane, silicon (Si) is also detected, indicating the successful incorporation of amino-functionalized mesoporous silica. Additionally, the nitrogen content increases compared to the pure PVDF membrane, owing to the amino groups present in the mesoporous silica. For the Am-MSN_2_/PVDF membrane, the silicon content further increases, reflecting the higher concentration of Am-MSNs added. However, an intriguing observation is that the nitrogen content in the Am-MSN_1_/PVDF membrane is slightly higher than in the Am-MSN_2_/PVDF membrane. This discrepancy may result from improved dispersion and the integration of 0.3 g of Am-MSNs in the Am-MSN_1_/PVDF membrane, which exposes more amino groups. Conversely, the addition of 0.6 g of Am-MSNs in the Am-MSN_2_/PVDF membrane could have led to partial aggregation or coverage of the particles, reducing the nitrogen exposure detected via EDS. These variations in elemental composition, driven by the incorporation and distribution of Am-MSNs, are expected to impact the overall performance of the membranes. These findings underscore the importance of optimizing the doping concentration and particle dispersion during the membrane fabrication process to achieve desirable functional properties [52,53].

### 3.2. Wettability Analysis of Membranes

**Figure 7:**
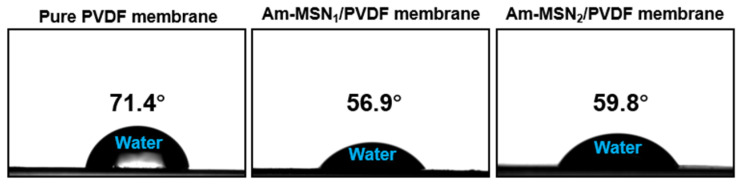
Water contact angles of the pure PVDF, Am-MSN_1_/PVDF, and Am-MSN_2_/PVDF membranes.

Wettability testing is crucial for evaluating membrane materials, as it provides insights into their hydrophilicity or hydrophobicity, predicts separation performance in practical applications, and assesses the effects of nanoparticle doping on surface properties, thereby aiding in the optimization of membrane functionality. As shown in Figure 7, the pure PVDF membrane, characterized by inherent hydrophobicity, exhibits a relatively high water contact angle in air, approximately 71.4°. This is attributed to the low surface energy of PVDF, which minimizes interactions between its surface and water molecules, resulting in pronounced hydrophobic behavior. With the incorporation of Am-MSNs, the water contact angle of the PVDF membranes decreases, signifying an enhancement in hydrophilicity. Specifically, the air–water contact angles for the Am-MSN_1_/PVDF and Am-MSN_2_/PVDF membranes are 56.9° and 59.8°, respectively. This improvement is primarily driven by the introduction of hydrophilic functional groups, such as the amino (-NH_2_) and hydroxyl (-OH) groups, which strengthen interactions between the membrane surface and water molecules. Additionally, the uniform distribution of Am-MSNs within the membranes not only enhances surface roughness but also creates additional water channels. The synergistic effects of the mesoporous structure and hydrophilic functional groups in Am-MSNs significantly contribute to the improved hydrophilicity [54]. Increased surface roughness provides more contact points for water molecules, thereby enhancing the affinity between the membrane and water [55]. These findings highlight the potential of Am-MSN incorporation to improve membrane wettability and overall performance.

### 3.3. Mechanical Performance Analysis of Membranes

**Figure 8:**
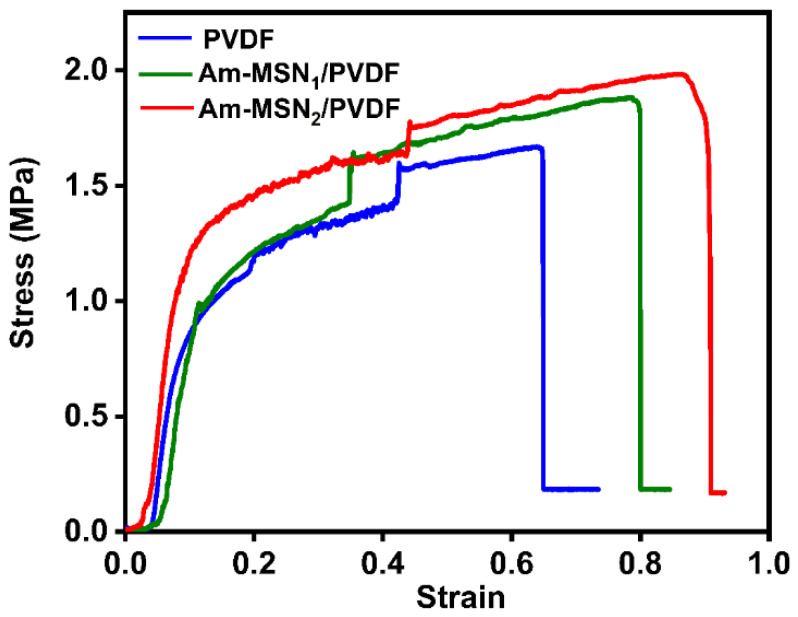
Representative stress–strain curves of the pure PVDF membrane, the Am-MSN_1_/PVDF membrane, and the Am-MSN_2_/PVDF membrane.

Figure 8 shows the stress–strain curves of the pure PVDF, Am-MSN_1_/PVDF, and Am-MSN_2_/PVDF membranes, illustrating the relationship between stress and strain (dimensionless) for these materials. The pure PVDF membrane demonstrates typical polymer behavior under strain, beginning with an initial linear phase (elastic deformation) and reaching a yield point at approximately 1.5 MPa stress and 0.3 strain. Beyond the yield point, the curve flattens, indicating limited ductility and rapid fracturing after yielding, which are characteristics of low tensile strength. Incorporating Am-MSN_1_ significantly enhances the mechanical properties of the PVDF membrane. The yield stress of the Am-MSN_1_/PVDF membrane increases by approximately 20% compared to the pure PVDF membrane, reflecting improved tensile strength. This enhancement is attributed to the effective dispersion of Am-MSNs and the strengthening of interfacial interactions within the membrane matrix. Beyond the yield point, the stress continues to increase slightly, suggesting improved rigidity and ductility due to the reinforcing effects of the nanoparticles. With the incorporation of twice the normal Am-MSN content, the Am-MSN_2_/PVDF membrane achieves the highest mechanical performance. The yield stress reaches 2.0 MPa, representing a 33% improvement over the pure PVDF membrane, while also exhibiting the highest yield strain among the three membranes. This indicates greater deformation capacity before transitioning to plastic deformation. Additionally, the modest stress increase beyond the yield point highlights the membrane’s balanced properties of high strength, ductility, and toughness. In summary, the incorporation of Am-MSNs markedly improves the mechanical properties of PVDF membranes. Both Am-MSN_1_/PVDF and Am-MSN_2_/PVDF membranes exhibit increased yield stress and yield strain, indicating enhanced strength and ductility. These improvements enable the membranes to endure higher stresses and deformations, making them more robust and suitable for separation operations under demanding conditions [56].

### 3.4. Separation Performance Analysis of Membranes

**Figure 9:**
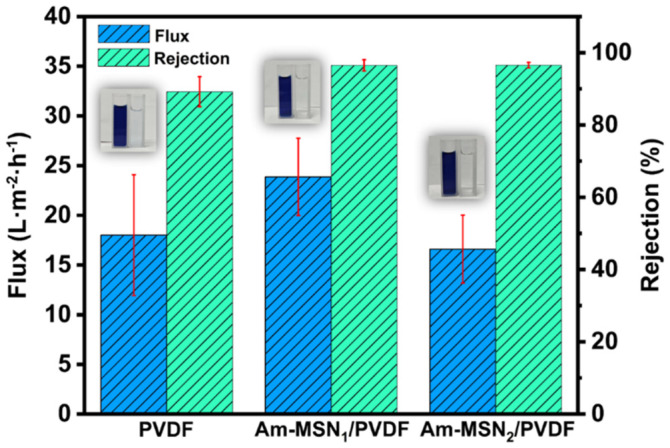
The dye wastewater flux and RB5 rejection of the pure PVDF, Am-MSN_1_/PVDF, and Am-MSN_2_/PVDF membranes. The inset in the figure presents a digital photographic comparison of the membrane before and after the removal of RB5.

Figure 9 illustrates the separation flux and dye rejection rates of the pure PVDF membrane, the Am-MSN_1_/PVDF membrane, and the Am-MSN_2_/PVDF membrane. The incorporation of Am-MSNs leads to an increase in separation flux, with the water flux of the Am-MSN_1_/PVDF membrane rising from 17 L m^−2^ h^−1^ for the pure PVDF membrane to 23.87 L m^−2^ h^−1^. This improvement is attributed to the hydrophilic nature of the Am-MSNs, which optimizes the membrane pore structure. During the coagulation process, these nanoparticles accelerate the exchange rate between the polymer matrix and the coagulation bath, facilitating the growth of finger-like pores. The resulting pore structure, with larger pore sizes, enhances the membrane’s permeability. The incorporation of Am-MSNs not only improves the surface hydrophilicity of the membrane but also introduces additional water channels, further boosting permeability. In the case of the Am-MSN_1_/PVDF membrane, the nanoparticles create extra pathways for water transport, significantly enhancing separation performance. Furthermore, the synergistic interaction between Am-MSNs and PVP enhances hydrophilicity, promoting efficient water passage through the membrane. However, the excessive addition of Am-MSNs can adversely affect performance. For the Am-MSN_2_/PVDF membrane, the water flux decreases to 16 L m^−2^ h^−1^. This decline is likely due to the aggregation of excess Am-MSNs, which can partially block or clog membrane pores, thereby restricting water flow and reducing permeability. These findings underscore the importance of optimizing the concentration of Am-MSNs to achieve a balance between improved permeability and minimal pore obstruction [57,58].

**Scheme 2:**
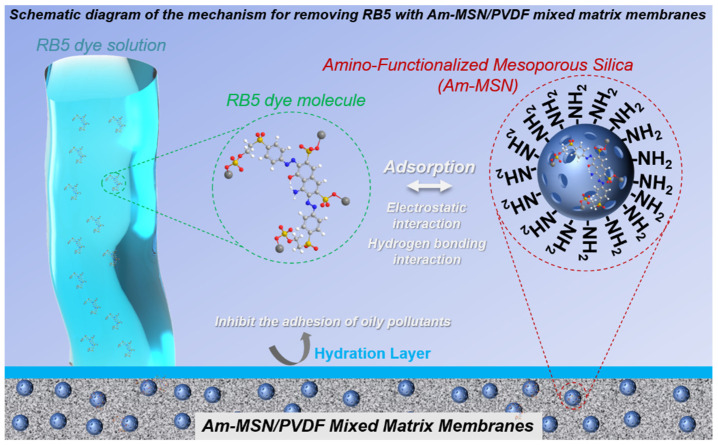
A schematic diagram illustrating the mechanism for removing the RB5 dye using Am-MSN/PVDF mixed matrix membranes.

RB5 is a commonly used organic dye extensively applied in the textile and dyeing industries. However, such dyes pose significant environmental challenges due to their high toxicity, carcinogenicity, and poor biodegradability, contributing to severe water pollution [59]. PVDF membranes, being inherently hydrophobic, exhibit limited affinity for both water and dye molecules like RB5. For polar, water-soluble dye molecules, strongly hydrophobic membranes are often ineffective in adsorbing or intercepting molecules with polar functional groups. In the case of the pure PVDF membrane, its pronounced hydrophobicity leads to the strong repulsion of RB5 molecules, causing the dye to accumulate on the membrane surface. This accumulation results in pore blockage, which significantly reduces both flux and rejection capacity. As shown in the figure, the pure PVDF membrane achieves a rejection rate of 89.20% for RB5. In contrast, the incorporation of Am-MSNs improves the hydrophilicity of the membranes, leading to the formation of a “hydration layer” on the surface. This hydration layer prevents oily pollutants from approaching the membrane directly, thereby minimizing the accumulation of oily pollutants within the pores and reducing flux decline. Moreover, the mesoporous structure of Am-MSNs within the membrane matrix provides abundant adsorption sites for dye molecules, thereby increasing the membrane’s specific surface area. The amino groups can form hydrogen bonds with the sulfonic acid groups in the dye molecules, while the protonated amino cations interact with the dye molecules through electrostatic attraction, which enhances the adsorption of the RB5 dye (Scheme 2). Consequently, the Am-MSN_1_/PVDF and Am-MSN_2_/PVDF membranes achieve enhanced RB5 rejection rates of 96.50% and 96.57%, respectively. Although the addition of 0.6 g of Am-MSNs in the Am-MSN_2_/PVDF membrane leads to a decrease in flux, its rejection rate remains high. This performance is attributed to the role of Am-MSNs within the membrane matrix, serving as physical adsorption sites for dye molecules and thereby enhancing dye rejection capabilities. These findings highlight the potential of Am-MSN/PVDF mixed matrix membranes to address challenges in wastewater treatment through improved dye rejection performance.

### 3.5. Static Adsorption Capacity Analysis of Membranes

**Figure 10:**
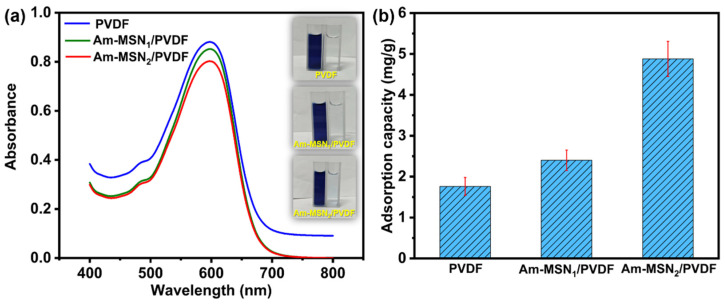
(**a**) UV spectra of permeate and (**b**) adsorption capacities of the pure PVDF, Am-MSN_1_/PVDF, and Am-MSN_2_/PVDF membranes. The inset in the figure provides a digital photographic comparison of the membrane before and after static adsorption of RB5.

Static adsorption capacity testing provides critical insights into the interaction between membrane materials and dye molecules. A higher adsorption capacity indicates stronger affinity between the membrane and dye molecules, which is essential for effective dye separation. Membrane separation performance is influenced not only by physical barrier effects but also by adsorption interactions with dye molecules. Figure 10a presents the static adsorption curves for RB5 on the pure PVDF membrane, the Am-MSN_1_/PVDF membrane, and the Am-MSN_2_/PVDF membrane, while Figure 10b details the corresponding adsorption capacities. The absorbance curves for all three membranes at different wavelengths exhibit a prominent peak at 597 nm, which is characteristic of the RB5 dye. Despite similar absorbance profiles at this wavelength, the membranes differ significantly in their adsorption capacities. The pure PVDF membrane exhibits an adsorption capacity of 1.76 mg g^−1^, reflecting its weak affinity for RB5 due to limited adsorption sites and low surface area [60]. In contrast, the Am-MSN_1_/PVDF membrane demonstrates an improved adsorption capacity of 2.4 mg g^−1^. This enhancement is attributed to the addition of 0.3 g of Am-MSNs, which increases the effective surface area and provides additional adsorption sites through their porous structure, facilitating dye molecule attachment. The Am-MSN_2_/PVDF membrane achieves the highest adsorption capacity of 4.88 mg g^−1^, indicating that the incorporation of 0.6 g of Am-MSNs further enhances adsorption performance [61]. The higher Am-MSN content introduces more micropores and surface-active sites, enabling the membrane to adsorb a greater number of dye molecules [62]. This improvement aligns with the enhanced dye rejection rates observed in Figure 9, corroborating the effectiveness of Am-MSNs as a functional additive for improving the adsorption and separation performance of PVDF membranes.

## 4. Conclusions

In conclusion, Am-MSN/PVDF mixed matrix membranes were successfully fabricated using the NIPS method for the efficient removal of RB5. Surface composition and microstructure analyses confirmed the successful preparation of the membranes. The incorporation of Am-MSNs enhanced the specific surface area and porosity of the PVDF membrane, while optimizing its pore distribution and morphology. These structural modifications contributed to a significant increase in porosity. Additionally, the enhanced hydrophilicity of the Am-MSN/PVDF mixed matrix membranes further improved permeation flux. The mesoporous structure of Am-MSNs also enhanced the mechanical strength of the membranes. Importantly, the high specific surface area and amino groups of Am-MSNs facilitated interactions with RB5 molecules via hydrogen bonding, electrostatic attraction, and physical adsorption, leading to improved dye rejection efficiency. The synergistic effects of the mesoporous structure and amino groups significantly enhanced both the efficiency and stability of the Am-MSN/PVDF mixed matrix membranes. This makes them a promising solution for the treatment of dye-contaminated wastewater. The study demonstrates that the Am-MSN/PVDF mixed matrix membranes offer a sustainable and efficient approach to addressing dye pollution in industrial wastewater.

## Figures and Tables

**Table 1:** Detailed preparation conditions of the pure PVDF and Am-MSN/PVDF mixed matrix membranes.

Membrane Samples	PVDF (g)	PVP (g)	DMF (g)	Am-MSN (g)
PVDF	11	4	35	0
Am-MSN_1_/PVDF	11	4	35	0.3
Am-MSN_2_/PVDF	11	4	35	0.6

**Table 2:** The melting temperature and crystallinity of the pure PVDF, Am-MSN_1_/PVDF, and Am-MSN_2_/PVDF membranes.

Sample	Melting Temperature (°C)	Crystallinity (%)
Pure PVDF	167.8	45.2
Am-MSN_1_/PVDF	168.5	47.3
Am-MSN_2_/PVDF	169.3	48.1

**Table 3:** The porosity of the pure PVDF, Am-MSN_1_/PVDF, and Am-MSN_2_/PVDF membranes.

Membranes	PVDF	Am-MSN_1_/PVDF	Am-MSN_2_/PVDF
Porosity (%)	35.8	69.7	68.7

**Table 4:** The semi-quantitative elemental composition of the pure PVDF, Am-MSN_1_/PVDF, and Am-MSN_2_/PVDF membranes.

Sample	C	N	O	F	Si	Total	Si/F
Pure PVDF	55.94	1.86	3.04	38.15	0	100.00	0
Am-MSN_1_/PVDF	52.59	3.30	3.75	39.77	0.60	100.00	1.51
Am-MSN_2_/PVDF	53.83	2.54	4.12	38.59	0.91	100.00	2.36

## Data Availability

The data that support the findings of this study are available from the corresponding author upon reasonable request.
